# Cell-Dependent Pathogenic Roles of Filamin B in Different Skeletal Malformations

**DOI:** 10.1155/2022/8956636

**Published:** 2022-07-04

**Authors:** Huixiao Wu, Yanzhou Wang, Xinyu Chen, Yangyang Yao, Wanyi Zhao, Li Fang, Xiaoqing Sun, Ning Wang, Jie Jiang, Ling Gao, Jiajun Zhao, Chao Xu

**Affiliations:** ^1^Department of Endocrinology and Metabolism, Shandong Provincial Hospital, Cheeloo College of Medicine, Shandong University, Jinan, 250021 Shandong, China; ^2^Department of Endocrinology and Metabolism, Shandong Provincial Hospital Affiliated to Shandong First Medical University, Jinan, 250021 Shandong, China; ^3^Institute of Endocrinology, Shandong Academy of Clinical Medicine, Jinan, 250021 Shandong, China; ^4^Shandong Clinical Medical Center of Endocrinology and Metabolism, Jinan, 250021 Shandong, China; ^5^Department of Pediatric Orthopedics, Shandong Provincial Hospital Affiliated to Shandong First Medical University, Jinan, 250021 Shandong, China

## Abstract

Mutations of filamin B (*FLNB*) gene can lead to a spectrum of autosomal skeletal malformations including spondylocarpotarsal syndrome (SCT), Larsen syndrome (LRS), type I atelosteogenesis (AO1), type III atelosteogenesis (AO3), and boomerang dysplasia (BD). Among them, LRS is milder while BD causes a more severe phenotype. However, the molecular mechanism underlying the differences in clinical phenotypes of different *FLNB* variants has not been fully determined. Here, we presented two patients suffering from autosomal dominant LRS and autosomal recessive vitamin D-dependent rickets type IA (VDDR-IA). Whole-exome sequencing revealed two novel missense variants in *FLNB*, c.4846A>G (p.T1616A) and c.7022T>G (p.I2341R), which are located in repeat 15 and 22 of filamin B, respectively. The expression of FLNB^I2341R^ in the muscle tissue from our LRS patient was remarkably increased. And in vitro studies showed that both variants led to a lack of filopodia and accumulation of the mutants in the perinuclear region in HEK293 cells. We also found that c.4846A>G (p.T1616A) and c.7022T>G (p.I2341R) regulated endochondral osteogenesis in different ways. c.4846A>G (p.T1616A) activated AKT pathways through inhibiting SHIP2, suppressed the Smad3 pathway, and impaired the expression of Runx2 in both Saos-2 and ATDC5 cells. c.7022T>G (p.I2341R) activated both AKT and Smad3 pathways and increased the expression of Runx2 in Saos-2 cells, while in ATDC5 cells it activated AKT pathways through inhibiting SHIP2, suppressed the Smad3 pathway, and reduced the expression of Runx2. Our study demonstrated the pathogenic mechanisms of two novel *FLNB* variants in two different clinical settings and proved that *FLNB* variants could not only directly cause skeletal malformations but also worsen skeletal symptoms in the setting of other skeletal diseases. Besides, *FLNB* variants differentially affect skeletal development which contributes to clinical heterogeneity of FLNB-related disorders.

## 1. Introduction

Filamins are a family of large dimeric actin-binding proteins which crosslink actin cytoskeleton filaments to exert crucial mechanotransduction and signaling functions in tissue morphogenesis [1]. There are three isoforms in the filamin family, respectively, filamin A (FLNA), B (FLNB), and C (FLNC). Mutations in *FLNA* and *FLNC* lead to a spectrum of hereditary diseases, including circulatory system, central nervous system, and skeletal system [2–4]. Mutations in *FLNB*, however, were solely found in skeletal diseases [5–7], which strongly suggested its crucial role in the development of skeletal system. Till now, mutations in the *FLNB* gene were mainly identified in five human skeletal disorders: spondylocarpotarsal syndrome (SCT; OMIM:272460), Larsen syndrome (LRS; OMIM:150250), type I atelosteogenesis (AO1; OMIM:108720), type III atelosteogenesis (AO3; OMIM:108721), and boomerang dysplasia (BD; OMIM:112310). All of them have overlapping clinical phenotypes, among which LRS is milder while BD causes a more severe phenotype. Missense mutations and small in-frame deletions within *FLNB* are the causative variants for LRS, while only nonsense mutations in *FLNB* have been reported in SCT [8]. LRS is an autosomal dominant osteochondrodysplasia characterized by large-joint dislocations, supernumerary ossification centers of carpal or tarsal bones, and characteristic craniofacial abnormalities including hypertelorism, prominence of the forehead, a depressed nasal bridge, and a flattened midface [9]. In recent years, our study has found several *FLNB* variants in other skeletal diseases except five mentioned above together with other pathogenic variants and we found that these patients often presented more severe symptoms than other patients who did not carry *FLNB* variants. However, further studies are required to establish its full clinical importance.

FLNB contains an actin-binding domain (ABD) consisting of two calponin homology domains (CH1 and CH2) forming a canonical F-actin-binding domain at the N-terminus, followed by 24 *β*-pleated sheet immunoglobulin- (Ig-) like repeats which are connected by two flexible hinge regions (H1 and H2) [10]. Up to date, there have been more than 150 variants in *FLNB* gene reported in the Human Gene Mutation Database (HGMD). Several mechanisms have been proposed to explain the pathogenesis of *FLNB* variants in skeletal malformations [11], including delay of ossification in long bone growth plate [12], reduction of bone mineral density [13], disturbance of proliferation, differentiation, and apoptosis in chondrocytes [14–16], and hypomotility of osteoblast and chondrocyte [12, 15, 17, 18]. However, only a limited number of variants have been evaluated for the pathogenic function and most of them focused on nonsense variants associated with SCT. Few articles reported the pathogenic mechanisms of *FLNB* missense variants partly due to the clinical heterogeneity of FLNB-related diseases. What is more, most previous studies were performed using HEK293 cells, which cannot fully mimic the real condition affected by *FLNB* variants in skeletal tissues.

In this study, we investigated the pathogenesis of two novel *FLNB* missense variants through in vivo and in vitro experiments and compared their difference at cellular and molecular levels. The novel *FLNB* variant associated with LRS was c.7022T>G (p.I2341R), which was found in our LRS patient, and another was c.4846A>G (p.T1616A), which was identified in a male patient with VDDR-IA. And we explored potential pathogenic mechanism of novel variants in terms of protein expression, cellular trafficking, and expression of molecular markers involved in endochondral osteogenesis and found that *FLNB* variants regulated skeletal development by different mechanisms which contributed to clinical heterogeneity of FLNB-related disorders. Our findings provide compelling evidence for elucidating the molecular mechanisms and help to explain the clinical heterogeneity of *FLNB*-related skeletal disorders.

## 2. Patients and Methods

### 2.1. Editorial Policies and Ethical Considerations

This study has been approved by the Ethics Committee of Shandong Provincial Hospital Affiliated to Shandong University. The study protocol was in line with the Declaration of Helsinki (as revised in Brazil 2013). All clinical images of the patients have been approved to be published by the patients or their guardians. Written informed consent was received from all participants prior to inclusion in the study.

### 2.2. Patients

A detailed medical history as well as family history was obtained. Physical examination, laboratory detection, and X-rays were performed to confirm the diagnosis. Peripheral blood samples were obtained from the patients and their family members for genetic testing. Muscle tissues were obtained, respectively, from our LRS patient and a healthy individual with hip joint dislocation during their surgeries.

### 2.3. DNA Extraction and Whole-Exome Sequencing

Genomic DNA was isolated from peripheral blood leukocytes using the QIAamp DNA Mini Kit (Qiagen, Germany) following the manufacturer's instructions. As for WES and subsequent Sanger sequencing for validation, we followed the methods of Wu et al. [19]. Genes and proteins were described according to the Human Genome Variation Society (HGVS) nomenclature guideline.

### 2.4. Bioinformatic Analysis

The bioinformatic analysis of *FLNB* (Gene ID: 2317, RefSeq: NM_001164317.2) variants was performed by two software tools to predict the potential pathogenic effects: MutationTaster (http://www.mutationtaster.org/), PolyPhen-2 (http://genetics.bwh.harvard.edu/pph2/), and PROVEAN (http://provean.jcvi.org) to predict disease-causing effects of the mutation. Furthermore, PyMOL software was used for visualizing the spatial structure and altered residues of the protein model.

### 2.5. Plasmid Construction

Full length of the major transcript of human *FLNB* was synthesized and cloned into the transient overexpression vector GV141 (GeneChem, China), using the restriction enzymes XhoI and BamHI (NEB, USA). c.4846A>G and c.7022T>G mutation sites were generated by site-directed mutagenesis (GeneChem, China). The detailed protocol used for site-directed mutagenesis is available upon request. The entire coding sequences of all the constructs were verified using sequencing.

### 2.6. Cell Culture and Transfection

HEK293 cells (National Collection of Authenticated Cell Cultures, Shanghai, China) were grown in DMEM (Gibco BRL, Gaithersburg, MD, USA) containing 10% fetal bovine serum at 37°C in 5% CO_2_ and 95% air. ATDC5 cells (National Collection of Authenticated Cell Cultures, Shanghai, China) were grown in DMEM/F-12 (Gibco BRL, Gaithersburg, MD, USA) containing 10% fetal bovine serum at 37°C in 5% CO_2_. Saos-2 cells (National Collection of Authenticated Cell Cultures, Shanghai, China) were grown in McCoy's 5A (Gibco BRL, Gaithersburg, MD, USA) containing 10% fetal bovine serum at 37°C in 5% CO_2_. Cells were seeded in a 6-well plate prior to transfection, and when they reached about 70% confluent, they were transfected with constructed FLNB-GV141 overexpression vector or empty GV141 vector using the Lipofectamine 3000 Transfection Kit (Invitrogen, USA). Transfection was performed for 8-10 h with 2.5 *μ*g plasmid per well, and cells were collected after transfection for 24-48 h.

### 2.7. Immunoblot Analysis

HEK293 cells were rinsed with cold PBS, and whole cell lysates were lysed in radioimmunoprecipitation assay (RIPA) buffer supplemented with protease and phosphatase inhibitors. Cell protein lysates (40 *μ*g) were separated using 10% SDS-PAGE and transferred onto polyvinylidene fluoride membranes (Millipore). The membranes were blocked with TBST containing 5% slim milk for 1 h at room temperature and then incubated with primary antibodies against Flnb (1 : 1000, Abcam), Ship2 (1 : 2000, Abcam), p-PI3K (1 : 1000, Cell Signaling Technology), PI3K (1 : 1000, PTM Biolab), p-Akt308/473 (1 : 1000, Cell Signaling Technology), Akt (1 : 1000, Abcam), p-Smad3 (1 : 1000, Cell Signaling Technology), Smad3 (1 : 1000, Cell Signaling Technology), Runx2 (1 : 1000, Cell Signaling Technology), Flag (1 : 1000, ProteinTech), actin (1 : 7500, ProteinTech), and GAPDH (1 : 7500, ProteinTech) overnight at 4°C and then were washed three times (10 minutes each time) after which they were incubated with secondary antibodies for 1 h at room temperature. The membranes were incubated with HRP-labeled secondary antibodies at room temperature for 1 h, and Immobilon Western HRP Substrate Peroxide Solution (Millipore, USA) was used for membrane development. The membrane was developed by Amersham Imager 680. And densitometry was performed by AlphaView software.

### 2.8. Immunofluorescence Assay

Forty-eight hours after transfection, cells were seeded on glass coverslips and then fixed with 4% paraformaldehyde, permeabilized with 0.3% Triton X-100, and blocked for 1 h in 2% BSA. Immunostaining was conducted with mouse anti-actin antibody (1 : 1000, ProteinTech) and rabbit anti-Flag antibody (1 : 200, ProteinTech) overnight at 4°C. Goat anti-mouse IgG (H+L) Alexa Fluor Plus 555 and goat anti-rabbit IgG (H+L) Alexa Fluor Plus 488 secondary antibodies (Invitrogen, USA) were used at 1 : 1000 at room temperature for 1 h. The cell nuclei were stained with DAPI (6-diamidino-2-phenylindole). Protein localization was observed by fluorescence microscopy (Carl Zeiss, Germany).

### 2.9. Statistical Analysis

Statistical analysis was performed by SPSS 19.0 software package (SPSS Inc., Chicago, IL, USA). The Kolmogorov-Smirnov test was used to determine the distribution of continuous variables. Continuous variables with normal distribution were given as mean ± SD and compared by independent samples Student's *t*-test while those with nonnormal distribution were given as median (25th, 75th percentiles) and compared by the Mann–Whitney *U* test. *N* = 3 means three biological replicates. *p* value < 0.05 was considered statistically significant.

## 3. Results

### 3.1. Clinical Features

#### 3.1.1. Family 1

**Figure 1:**
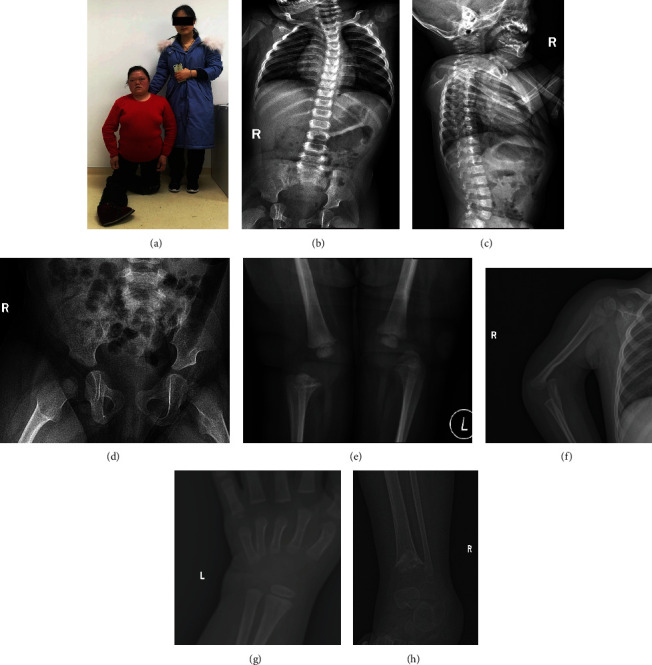
Clinical images of the LRS patient. (a) Patient II-3 (the LRS proband's mother) showed bilateral talipes equinovarus, multiple joint dislocation, and characteristic face of LRS (widely spaced eyes and depressed nasal bridge). (b) The spine X-ray of the LRS proband demonstrated that the thoracic spine was curved to the left and (c) the cervical curvature turned straight. (d) Pelvis radiograph of the LRS proband indicated that her acetabulum shaped like a “shallow dish” leading to bilateral dislocation of the hip joint. (e) Radiograph of both knee joints showed bilateral dislocation of the knee joint. (f) Right upper limb X-rays presented elbow joint dislocation. (g) Left wrist X-rays found that no ossification center appeared. (h) Radiograph of right ankle joints showed epiphyseal closure of distal tibia and basitarsi have disorganized bone architecture.

The proband, a 5-month-old Chinese baby girl, was first admitted to our hospital for bilateral talipes equinovarus and multiple joint dislocations at birth. The investigation of family history revealed that her mother, aunt, and grandmother had congenital talipes equinovarus and multiple joint dislocation similar to the patient. And her mother showed a characteristic face of LRS (widely spaced eyes and depressed nasal bridge). As the proband was only five-month-old at the first diagnosis, the carpal bones were not formed yet. So supernumerary ossification centers of carpal or tarsal which is a typical clinical phenotype of LRS were not seen in our proband temporarily. The serum biochemical and hormone assays of the proband at the first clinical examination showed increased alkaline phosphatase (579 U/L, normal range: 23-140 U/L) with normal serum calcium (2.6 mmol/L, normal range: 2.2-2.7 mmol/L) and phosphate (1.95 mmol/L, normal range: 1.29-2.26 mmol/L). Radiographs showed mild dorsal scoliosis, and bilateral dislocations of hip, knee, and elbow joints (Figure 1).

#### 3.1.2. Family 2

The proband, a 32-year-old Chinese man, was first admitted to our hospital for refractory bone fractures, dental dysplasia, and scoliosis. He was only 140 cm at the first diagnosis (-5.45 SD). The serum biochemical and hormone assays at the first clinical examination showed decreased vitamin D, normal serum calcium, phosphate, parathyroid hormone, and increased alkaline phosphatase. Radiographs showed scoliosis and genu varum. There were no extraskeletal manifestations. His older sister had milder skeletal symptoms including short statue (-2 SD), genu varum, and occasional limb weakness.

### 3.2. Identification of Two Heterozygous FLNB Missense Mutations

**Figure 2:**
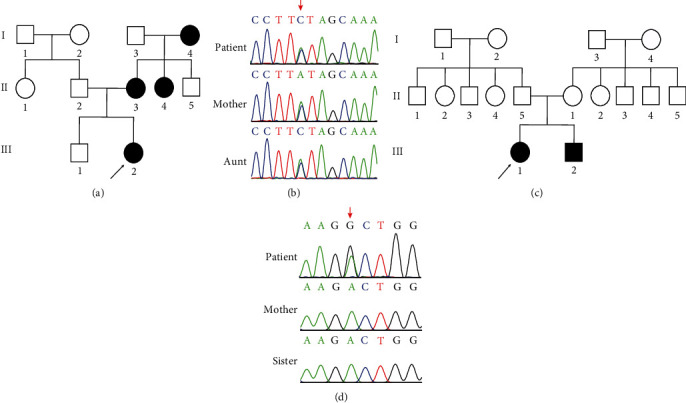
The pedigree of two families with novel *FLNB* variants (c.4846A>G and c.7022T>G). (a) Pedigree of a Chinese LRS family. Males and females are indicated by squares and circles. Affected individual is represented by filled symbols. The proband is represented by arrows. (b) Partial DNA sequence of the mutation site (c.7022T>G) in the *FLNB* gene. (c) Pedigree of a Chinese VDDR-IA family. Males and females are indicated by squares and circles. Affected individual is represented by filled symbols. The proband is represented by arrows. (d) Partial DNA sequence of the mutation site (c.4846A>G) in the *FLNB* gene.

To confirm the pathogenic gene of these two patients, they were subjected to whole-exome sequencing and detected variants were further confirmed via Sanger sequencing in their family members. We found that the proband of family 1 shared a novel heterozygous c.7022T>G (p.I2341R) variant with her mother and aunt (Figures 2(a) and 2(d)). The proband and his elder sister of family 2 carried a novel homozygous c.4846A>G (p.T1616A) variant besides disease-causing CYP27B1 variants (c.913delC/p.Q305Sfs∗7; c.125G>T/p.G42V) (Figures 2(c) and 2(d)). Both have not been reported in the HGMD, TOPMED, ExAC, and 1000 Genomes databases, indicating that the mutations we have found were novel and rare. c.4846A>G (p.T1616A) and c.7022T>G (p.I2341R) variants were located in the repeat 15 and 22 of filamin B, respectively, and substituted original amino acid without changing the length of the protein.

### 3.3. Bioinformatic Analysis

**Figure 3:**
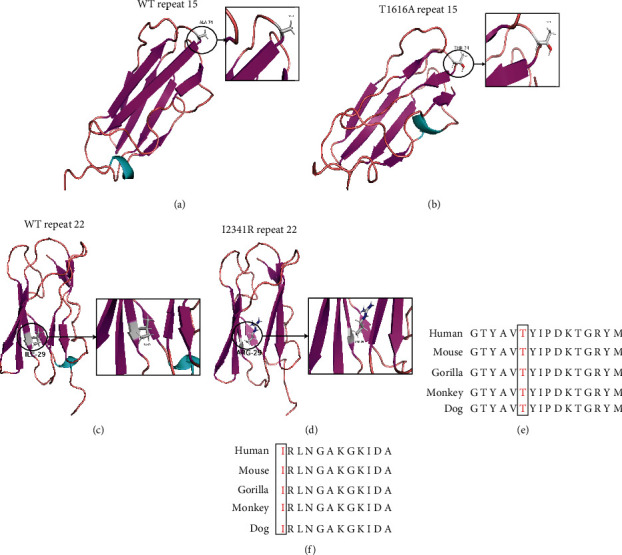
Protein structure prediction of wild-type and mutant FLNB repeat 15 and 22 and sequence alignments of mutated region. (a) Protein structure prediction of wild-type repeat 15. (b) Protein structure prediction of T1616A repeat 15. (c) Protein structure prediction of wild-type repeat 22. (d) Protein structure prediction of I2341R repeat 22. (e) Conservation analysis of T1616A variant via multiple sequence alignment. Changed amino acids are marked in red. (f) Conservation analysis of I2341R variant via multiple sequence alignment. Changed amino acids are marked in red.

To clarify the pathogenic mechanism of these two novel *FLNB* variants, we first performed bioinformatic analysis. Both missense variants affected highly conserved amino acids in diverse species by multiple sequence alignment (Figures 3(e) and 3(f)), highly suggesting they have disease-causing effects. c.7022T>G (p.I2341R) was strongly predicted to be pathogenic and deleterious using three online bioinformatic software—MutationTaster, PolyPhen-2, and PROVEAN, while c.4846A>G (p.T1616A) was predicted to be pathogenic by MutationTaster but benign through PolyPhen-2 and PROVEAN.

The protein model of mutant and wild-type repeat region of FLNB was built by I-TASSER automatically and visualized by PyMOL viewer. Both variants were predicted to disrupt the structure of FLNB. c.4846A>G (p.T1616A) changed the structure of nearby *β*-sheet as well as the N-terminal alpha-helix (Figures 3(a) and 3(b)) and c.7022T>G (p.I2341R) aborted the structure of both N-terminal alpha-helices and C-terminal *β*-sheets (Figures 3(c) and 3(d)), which may affect the modification of FLNB protein or its interaction with other proteins.

### 3.4. Expression Pattern of Wild-Type and Mutant FLNB Proteins in Patient's Muscle Tissue and HEK293 Cells

**Figure 4:**
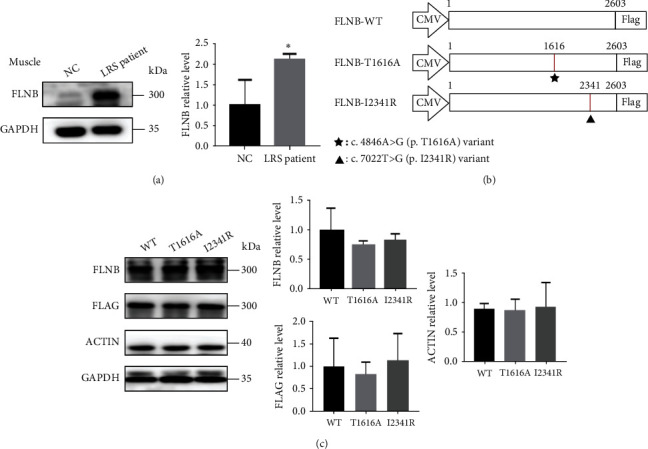
Expression pattern of WT and mutant FLNB proteins in patient's muscle tissue and HEK293 cells. (a) The lysates of muscle tissues from normal control and our LRS proband were fractioned on 10% SDS-PAGE and analyzed by immunoblotting with anti-FLNB antibody. (b) Schematic diagrams of full-length and mutant *FLNB* vectors transfected into HEK293 cells. The numbers represent the mutation site in FLNB. The mutations were labeled with stars and triangles, respectively. (c) Whole HEK293 cell lysates from transiently transfected with WT or mutant *FLNB* plasmids were fractioned on 10% SDS-PAGE and analyzed by immunoblotting with anti-FLNB and anti-actin antibodies (*N* = 3). Quantitative analysis of FLNB, Flag, and actin levels was shown by the mean percentage ± SD normalized to GAPDH levels (^∗^*p* < 0.05, ^∗∗^*p* < 0.01, and ^∗∗∗^*p* < 0.001 by Student's *t*-test).

To further explore the pathogenic role of novel *FLNB* variants, we performed a series of experiments. First, we evaluated the expression pattern of FLNB in the muscle tissue from our LRS patient. And we found that the expression of FLNB^I2341R^ mutant was significantly increased compared with normal control (Figure 4(a)). Then, we tested the overall expression level of WT and mutant FLNB in HEK293 cells transfected with vectors expressing Flag-WT-FLNB (FLNB^WT^) or Flag-mutant-FLNB (FLNB^T1616A^ and FLNB^I2341R^) (Figure 4(b)). Immunoblot analysis showed that FLNB^WT^ was presented as a single band with an approximate molecular weight of 270 kDa and both variants situated at the same position with WT (Figure 4(c)). The expression of FLNB^T1616A^ and FLNB^I2341R^ showed no significant difference compared with FLNB^WT^.

### 3.5. Cellular Localization of Wild-Type and Mutant FLNB

**Figure 5:**
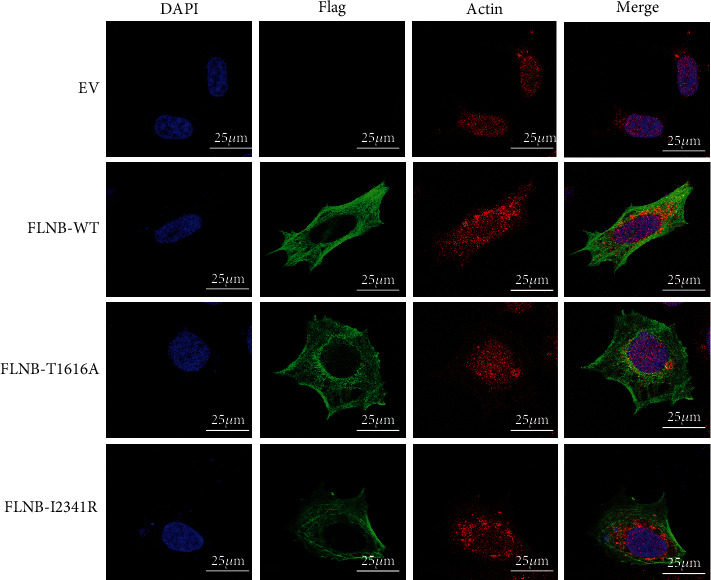
Subcellular localization analysis of wild-type and FLNB mutants in HEK293 cells. HEK293 cells were transfected with *FLNB* plasmids carrying WT-FLNB or mutated FLNB. 48 hours later, cells were fixed, permeabilized, and immunostained with the anti-Flag antibody (green) and anti-actin antibody (red). Nuclei were visualized by DAPI. The slides were visualized on fluorescence confocal microscopy (Leica, Germany). Scale bars represented 25 *μ*m. Original magnification: 400x.

As filamin B regulates the cytoskeleton network by crosslinking actin fibrils, then we tested whether novel mutations had influences on FLNB subcellular localization and their interaction with actin via immunofluorescence assay. Both variants did not affect the actin-binding domain of FLNB, so we hypothesized that mutant FLNB might still associate with actin. The results showed that wild-type FLNB expressed in HEK293 cells was evenly distributed within the cytoplasm presenting a fine cytoskeleton network and colocalized with actin, while FLNB^T1616A^- and FLNB^I2341R^-transfected cells showed a lack of filopodia and turned rounder which might have a negative effect on cell migration, and mutant FLNB proteins tended to accumulate in the perinuclear region together with actin, indicating an intracellular retention of mutant FLNB (Figure 5).

### 3.6. FLNB^T1616A^ and FLNB^I2341R^ Regulated the Expression of Runx2 through Different Mechanisms in Saos-2 Cells

**Figure 6:**
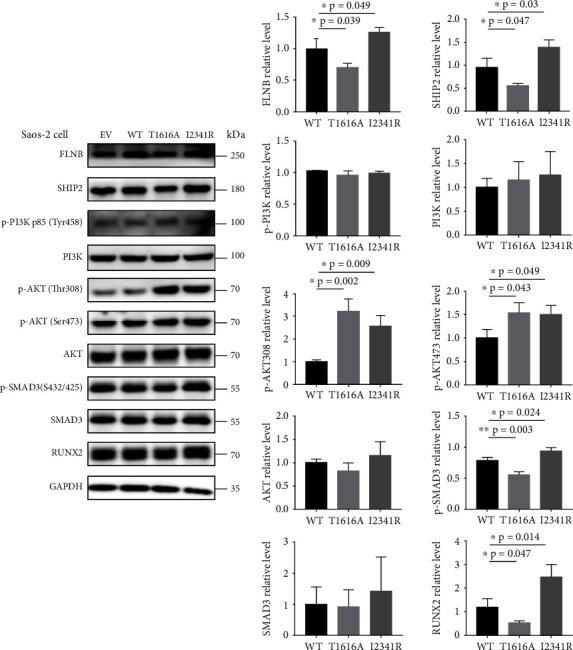
Expression patterns of wild-type and FLNB mutants and osteogenesis-related marker Runx2 as well as upstream signaling pathways in Saos-2 cells. Whole Saos-2 cell lysates from transiently transfected with WT or mutant FLNB plasmids were fractioned on 10% SDS-PAGE and analyzed by immunoblotting (*N* = 3). Quantitative analysis of FLNB, SHIP2, p-PI3K, t-PI3K, p-AKT, t-AKT, p-SMAD3, t-SMAD3, and Runx2 levels was shown by the mean percentage ± SD normalized to GAPDH levels (^∗^*p* < 0.05, ^∗∗^*p* < 0.01, and ^∗∗∗^*p* < 0.001 by Student's *t*-test).

To further evaluate the pathogenic mechanism of both *FLNB* variants, we examined the expression of the major osteogenesis marker Runt-related transcription factor 2 (Runx2) and its related signaling pathways using Western blot in a human osteosarcoma cell line Saos-2 which displays several osteoblastic features. In Saos-2 cells transfected with FLNB^T1616A^, the expression of FLNB was obviously decreased while those transfected with FLNB^I2341R^ had a significantly increased expression of FLNB (Figure 6), which was consistent with our in vivo results above. And the same trends were observed in the expression of Ship2, Runx2, and phosphorylated Smad3 with total Smad3 unchanged (Figure 6). Besides, the phosphorylated Akt was increased in both Saos-2 cells expressing FLNB^T1616A^ and FLNB^I2341R^, while the expression of PI3K and total Akt was not changed (Figure 6).

### 3.7. FLNB^T1616A^ and FLNB^I2341R^ Regulated the Expression of Runx2 through Similar Mechanisms in ATDC5 Cells

**Figure 7:**
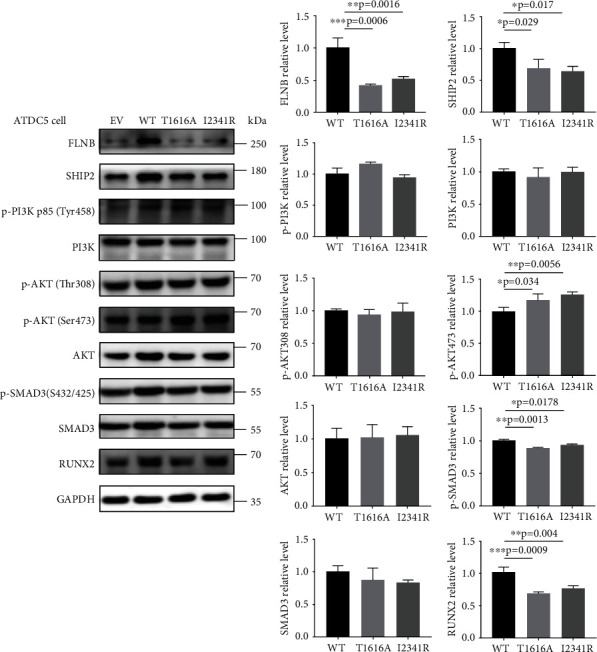
Expression patterns of wild-type and FLNB mutants and osteogenesis-related marker Runx2 as well as upstream signaling pathways in ATDC5 cells. Whole ATDC5 cell lysates from transiently transfected with WT or mutant FLNB plasmids were fractioned on 10% SDS-PAGE and analyzed by immunoblotting (*N* = 3). Quantitative analysis of FLNB, SHIP2, p-PI3K, t-PI3K, p-AKT, t-AKT, p-SMAD3, t-SMAD3, and Runx2 levels was shown by the mean percentage ± SD normalized to GAPDH levels (^∗^*p* < 0.05, ^∗∗^*p* < 0.01, and ^∗∗∗^*p* < 0.001 by Student's *t*-test).

Besides performing functional study in Saos-2 cells, we also explored the pathogenic role of both *FLNB* variants in the ATDC5 cell line which was derived from mouse teratocarcinoma cells and characterized as a chondrogenic cell line. Different from the results of Saos-2 cells, the expression of FLNB was significantly decreased in both ATDC5 cells transfected with FLNB^T1616A^ and FLNB^I2341R^ (Figure 7). And the same trends were observed in the expression of Ship2, Runx2, and Smad3 (Figure 7). Besides, phosphorylated Akt was significantly increased in ATDC cells expressing FLNB^T1616A^ and FLNB^I2341R^ without changing the phosphorylation level of PI3K (Figure 7).

## 4. Discussion

Here, we identified two novel *FLNB* variants in two Chinese patients, one of which was diagnosed with LRS and the other was confirmed to have VDDR-IA. In silico analysis suggested a strong possibility for pathogenic significance of both variants. And functional studies confirmed that both novel variants could contribute to the pathogenesis of their skeletal deformities through impairing protein expression, protein subcellular localization, and endochondral osteogenesis but they regulated osteogenesis in totally different ways, which accounted for the clinical heterogeneity of FLNB-related diseases. Moreover, we made the first attempt to analyze the expression of FLNB in our patient's tissue and in Saos-2 and ATDC5 cells to fully reproduce FLNB-induced pathological changes in skeletal development. Thus, bioinformatics together with functional characterization provided strong evidence for the pathogenic role of our new-found *FLNB* variants.

Mutations in *FLNB* cause a spectrum of skeletal dysplasia, from mild types, like SCT and LS, to severe types, such as AO1, AO3, and BD. SCT is characterized by premature carpal and tarsal fusion and blocking the vertebrae leading to spinal deformity [8]. BD is a perinatal lethal osteochondrodysplasia characterized by absence or underossification of the limb bones and vertebrae [10]. The manifestations of AO1 and AO3 fall in between those of LRS and BD [11, 12]. Diverse phenotypes in human FLNB-related disorders correlate with the impact of variants on protein expression, subcellular localization, and downstream molecules. Missense mutations and small in-frame deletions within FLNB are the causative mutations for LRS characterized by supernumerary carpal bones, large joint dislocations, spinal malformations, and characteristic facial malformations including prominent forehead, wide-spaced eyes, and depressed nasal bridge. Known variants leading to LRS are randomly located in the CH2 domain and domains near hinge regions including repeat 2, 5, 13, 14, 15, 16, 17, and 23 of FLNB. Recently, it has been showed that nonrandom clustering of LS-AO-BD disease-causing variants has at least two pathogenic mechanisms of generating these disease phenotypes, one of which relates to FLNB-actin-FLNA focal accumulation of the amount which was correlated with phenotype severity of LRS and the other possibly dysregulating the function of hinge 1 [20]. Yet how the maldistribution of FLNB leads to skeletal dysplasia is still uncertain.

**Figure 8:**
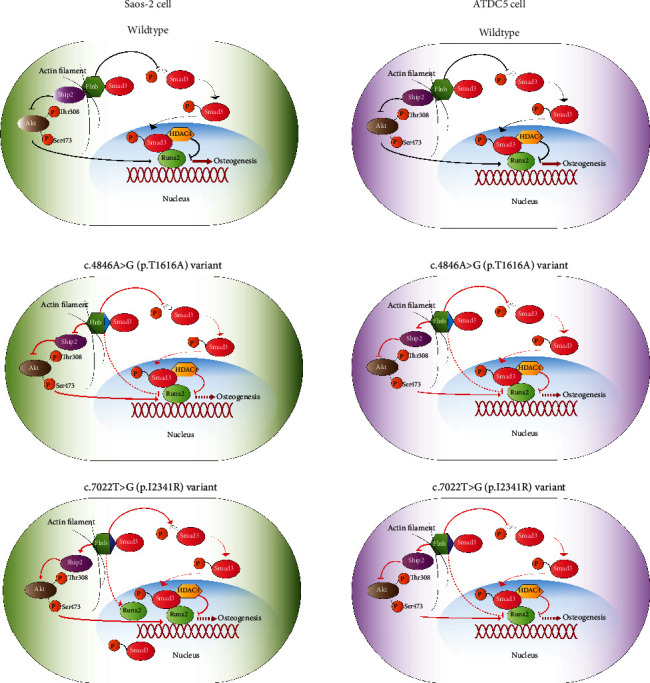
Schematic diagram of potential molecular mechanisms underlying the pathogenesis of two novel *FLNB* missense variants. Two novel *FLNB* variants caused two different skeletal malformations through regulating the expression and transcriptional activity of Runx2 in a cell-dependent way. In LRS, increased expression of Runx2 may promote osteogenesis and condensation, which further leads to supernumerary ossification and early closure of epiphyses. In VDDR, the expression of Runx2 was significantly impaired, thus worsening skeletal dysplasia of the patient. Affected signaling pathways were labeled in red.

Skeletogenesis is tightly controlled by transcription factors. Among them, Runt-related transcription factor 2 (Runx2), also known as CBFA1, AML3, or OSF2, is essential for osteoblast differentiation and chondrocyte maturation [21, 22]. To examine the effect of *FLNB* variants on osteogenesis process, we first transfected *FLNB* vectors into Saos-2 cells. The downregulation of Runx2 expressing FLNB^T1616A^ in our research demonstrated that c.4846A>G (p.T1616A) variant could aggravate skeletal manifestations of our VDDR proband by impairing osteogenesis, while the upregulation of Runx2 in cells expressing FLNB^I2341R^ observed could explain the early closure of epiphyses in our LRS proband. Smad3 could bind to FLNB protein preventing Smad3 from being phosphorylated [14]. When phosphorylated Smad3 entered the nucleus, they recruited histone deacetylase 4 (HDAC4) to inhibit Runx2 activity through forming a HDAC4–Smad3–Runx2 complex [23]. Interestingly, although the expression of Runx2 was increased in FLNB^I2341R^-expressing Saos-2 cells, the expression of phosphorylated Smad3 was also raised, indicating that the Runx2 activity was repressed and endochondral osteogenesis might also be altered (Figure 8).

The serine/threonine protein kinase Akt is one of the key intracellular transducers of bone anabolic signals. Akt promotes BMP2-induced osteoblast differentiation and enhances the function and transcriptional activity of Runx2 [24]. Fujita et al. found Runx2 and PI3K-Akt signaling were mutually dependent on each other in the regulation of osteoblast and chondrocyte differentiation and their migration [25]. And it has been recently demonstrated that Akt regulates osteoblast differentiation, at least in part, by enhancing the protein stability and transcriptional activity of Runx2 through regulation of ubiquitin/proteasome-mediated degradation of Smurf2 [26]. However, the precise molecular mechanism underlying the relationship between Runx2 and Akt is not well understood. Src homology 2 domain-containing inositol phosphatase 2 (SHIP2), which is encoded by inositol polyphosphate phosphatase-like 1 (INPPL1) gene, functions to dephosphorylate and negatively regulate intracellular phosphatidylinositol phosphate (PI(3,4,5)P3), a key second messenger of various intracellular signaling pathways including the AKT pathway. SHIP2 has been widely studied in insulin resistance [21, 27, 28], obesity [22], and cancer [29], and recently, it was reported that mutations in *INPPL1* cause opsismodysplasia, a rare autosomal recessive severe skeletal dysplasia [30, 31]. c.4846A>G (p.T1616A) was located in repeat 15 and c.7022T>G (p.I2341R) was located in repeat 22 of FLNB, which is the binding site of FLNB to inositol polyphosphate 5-phosphatase (SHIP2). So it was natural for us to speculate that c.7022T>G (p.I2341R) variant might cause LRS by impairing the function of SHIP2. And our results showed that the phosphorylated Akt was increased in both Saos-2 cells expressing FLNB^T1616A^ and FLNB^I2341R^ with PI3K and total Akt expression unchanged. But the expression of SHIP2 in FLNB^I2341R^ expressed Saos-2 cells, which seemed contradictory to the fact that SHIP2 was a negative regulator of the PI3K/AKT pathway, indicating that FLNB^I2341R^ might abort the function of SHIP2 through posttranslational mechanisms or regulate the PI3K/AKT pathway by some other factors except SHIP2 (Figure 8).

Runx2 also plays an important role in chondrogenesis, which is the prerequisite of endochondral osteogenesis. Akiyama et al. found that as ATDC5 cells condensed to cartilaginous nodules, the expression of Runx2 increased through the whole process until the end of chondrocytic maturation [29]. The cellular condensation of undifferentiated ATDC5 cells and subsequent process were inhibited when the dominant negative form of Runx2 was introduced to the cells. And Runx2 has been shown to be obligatory for both proliferation and differentiation of chondrocytes [32]. Thus, Runx2 also plays a positive role in the regulation of chondrogenesis. Previous studies have showed that FLNB participates in chondrocyte proliferation and differentiation in the growth plate through interaction with the cytoskeleton by binding to filamentous actin or through dimerization to crosslink actin fibrils [1, 15, 16, 33, 34]. So we next used ATDC5 cells to evaluate the pathogenic effects of *FLN*B variants on chondrogenesis. Interestingly, different from the results in Saos-2 cells, the expression of FLNB and Runx2 as well as SHIP2 and p-Smad3 in both ATDC5 cells expressing FLNB^T1616A^ and FLNB^I2341R^ was significantly decreased. From this point of view, both novel *FLNB* variants may impair cellular condensation of mesenchymal cells during embryonic skeletal development, which could explain the multiple joint dislocation of our LRS patient (Figure 8). Moreover, as in Saos-2 cells, the Akt pathway was also activated in both FLNB^T1616A^- and FLNB^I2341R^-expressing ATDC5 cells.

The main innovations of our study can be concluded as the following three points. First of all, although previous studies have explored the pathogenic mechanisms of *FLNB* missense variants, most of the experiments were performed using HEK293 cells due to their high transfection rate [17, 20]. However, as a kidney-derived cell line, HEK293 cannot fully mimic FLNB-induced pathological changes in skeletal development. Previous studies showed that autosomal-dominant mutations of FLNB leading to AO1/AO3/BD/LRS did not alter protein levels in HEK293 cells [8], but they ignored the possibility that FLNB might exert its function in a cell-dependent way, and as FLNB variants only led to skeletal diseases, it was more rational for researchers to perform in vitro studies in a bone-derived cell line rather than HEK293. Nevertheless, Saos-2 (human osteosarcoma cells) and ATDC5 (mouse teratocarcinoma cells) were, respectively, osteoblastic and chondrogenic cell lines, which were much more suitable for the study of FLNB in skeletal diseases. In fact, it turned out that Saos-2 and ATDC5 cells did differ from HEK293 cells. For example, the expression of FLNB was demonstrated not to be affected in HEK293 while significantly affected in Saos-2 and ATDC5. This result indicated that the physiological functions of FLNB were cell-type dependent, which partly explained why *FLNB* variants were only related to skeletal diseases. Secondly, our study is the first one to examine the expression level of FLNB in patient's tissue, which provide robust evidence for the study of pathogenesis of *FLNB* variants. Thirdly, up to date, there has been no report on the relationship between FLNB and SHIP2 in skeletal deformities. In our present study, we first showed that FLNB might regulate the expression of SHIP2 which is a newly discovered skeletal developmental regulator and further modulate downstream the PI3K/AKT pathway and Runx2 activity, which helps us to explain the pathogenesis underlying FLNB-related skeletal malformations.

There also existed some limitations in our study. First, we only chose two novel variants to perform functional analysis and the function of other novel *FLNB* variants needs to be further explored to determine the generalizability of our findings. Secondly, as in vitro cell models were still not enough to represent skeletal developmental process, we are now establishing a LRS mouse model to better understand the pathogenesis of new-found *FLNB* missense variants.

In summary, our study identified two novel heterozygous *FLNB* missense disease-causing variants in two different skeletal malformations and was the first to perform a confirmatory study of them in vivo and in vitro. Our data expanded the mutation spectrum of *FLNB* and promoted better understanding of the pathogenesis and the clinical heterogeneity of *FLNB*-related skeletal disorders. Moreover, the combination of molecular findings with clinical data would accelerate to understand pathogenic mechanisms and facilitate early diagnosis and management of the patients with FLNB-related skeletal disorders.

## Data Availability

All data included in this study are available upon request by contacting the corresponding author.
